# A retrospective analysis of the social determinants of health affecting stroke outcomes in a small hospital situated in a health professional shortage area (HPSA)

**DOI:** 10.1371/journal.pgph.0001933

**Published:** 2024-01-08

**Authors:** Evelyn B. Voura, Ynesse Abdul-Malak, Tabatha M. Jorgensen, Sami Abdul-Malak

**Affiliations:** 1 Crouse Neuroscience Institute, Crouse Health at Crouse Hospital, Crouse Medical Practice, Syracuse, New York, United States of America; 2 Department of Neuroscience and Physiology, State University of New York (SUNY) Upstate Medical University, Syracuse, New York, United States of America; 3 Department of Sociology and Anthropology, Colgate University, Hamilton, New York, United States of America; University of Bristol Department of Community Based Medicine: University of Bristol Population Health Sciences, UNITED KINGDOM

## Abstract

Where someone lives is a major determinant of population health. In the United States, people who live in Health Professional Shortage Areas are considered medically underserved and have a higher propensity for conditions such as stroke, hypertension, and diabetes. Our goal was to better understand the diverse needs of patients presenting to the Crouse Hospital emergency department with stroke symptoms. Crouse Hospital is a small community hospital located in a shortage area serving both urban and rural populations in and around Syracuse, New York. Despite its small size, Crouse Hospital quickly became a major comprehensive stroke center in Central New York. With this study we assessed the social factors affecting the stroke patient population in the community and compared these characteristics between those living in served and underserved areas. Informed by the social determinants of health framework, we analyzed 1731 incidents of stroke that occurred between January 2019 and January 2021, and observed that the circumstances associated with stroke varied by service category and race, with White patients and those from served areas having better stroke outcomes compared to those residing in underserved areas and those that were not White. Our analyses help us to understand the underlying factors influencing the observed disparities and allow us to move forward by implementing informed community-based interventions to decrease stroke incidence and improve post-stroke care. Using our example other small hospitals can enact similar strategies to address the social determinants affecting their patients to improve stroke outcomes in their region.

## Introduction

Stroke is one of the leading causes of death and serious long-term disability in the United States. According to the Centers for Disease Control and Prevention (CDC), it accounts for one death every 3.5 minutes [1]. The majority of strokes (80%) are avoidable if risk factors such as hypertension, diabetes, smoking, and high blood cholesterol are proactively addressed [2]. The diagnosis and effective treatment of these and other medical conditions correlates with reliable access to healthcare [3].

Despite national efforts to decrease the risk factors contributing to the high incidence of stroke, some populations and geographic areas with limited or no access to primary care services continue to have a greater incidence of stroke and the associated morbidity and mortality [2, 4, 5]. These “high burden” populations tend to be non-White [6]. Furthermore, studies demonstrate that underserved and racial minority populations are particularly vulnerable due to underlying social determinants of health (SDoH) that are structural in nature [5]. Consequently, the Black and Hispanic communities have higher mortality rates from stroke than their White community counterparts because of chronic conditions and social factors contributing to inherent disadvantages in these groups [1, 6, 7]. In fact, Black individuals are nearly twice as likely to have a first stroke and are more likely to die from a stroke than White individuals [1]. Racial minorities often have detrimental cardiovascular issues such as high blood pressure, blood glucose and blood cholesterol that go untreated due to being uninsured or underinsured, living in medically underserved areas (urban and rural) with limited access to routine health care, as well as other contributing systemic socioeconomic factors [2].

To prevent recurrent stroke, others highlighted that along with addressing chronic underlying medical conditions, it is necessary to enable patient access to care following discharge from the hospital despite the often scarcity of resources [8]. Notably, strategies focused on education for stroke prevention alone were not effective [9–11]. To make a difference, programs need to be tailored to the requirements of the underserved and/or racialized patient population, and to be sustainable, these patient-focused solutions should make the best use of available resources and personnel [12]. Ultimately, if access to basic health care is not established, targeting underlying risk factors such as diabetes and hypertension are not sufficient to make headway in stroke prevention [8].

Using a SDoH framework can help explain how pronounced disparities affect stroke outcomes. SDoH include factors such as a lack of health insurance, social isolation, low income and education, socioeconomic status, living in impoverished and/or rural areas, and living in regions with limited access to health care settings [13, 14]. These factors are not clinical or biological, but are mostly social and environmental, yet still have a profound effect on stroke outcomes with the affected populations having higher rates of stroke when compared to the general population. Among the most affected groups are Black women, the uninsured, obese individuals, and younger people (under 65 years of age) who visit the doctor less frequently. This is in part because the associated individuals tend to have a higher rate of undiagnosed hypertension, an important risk factor for stroke [15]. Furthermore, as the number of SDoH affecting a person increases, so does their risk of stroke [5]. Importantly, published work has documented that as the number of factors contributing to health disparities increase, enabling access to medications, such as statins to control blood cholesterol, can effectively help to mitigate the chance of stroke and incidences of heart attack among Black women and the uninsured [16, 17].

The objective of our study was to identify what aspects of stroke care should be emphasized within the Crouse Hospital patient community to further improve the quality of our stroke outcomes. As a first step, we worked to identify the key SDoH among the underserved and different racial groups in our care to determine how these might contribute to health disparities, thereby better informing our understanding of the medical issues affecting the consequences of their stroke. The importance of these social factors to stroke are significant and have led some to name stroke a “disease of disparities” [18–20]. While others have focused on personal risk factors such as smoking status with limited success, we hope that by studying the social factors affecting our patients, we will have a clearer picture of our patient population and the underlying health factors leading up to their stroke and influencing the success of their post stroke care [21–23]. To address these factors, here we document the results of our retrospective examination of served and underserved individuals among different racial groups who came to the Crouse Hospital emergency department (ED) with stroke symptoms between January 2019 and January 2021. The social factors we examined were structural for the most part, and not easily modified with lifestyle changes. For the purposes of our analysis, we assigned patients into served or underserved populations based on a metric provided by the United States Health Resources and Services Administration (HRSA). A number of assessments contribute to the derivation of the assigned number (detailed in the Methods) and a score indicates the area is underserved and therefore, a target for physician placement and additional funding by Medicare. In the end, our analyses helped us to identify ways we can easily modify our post-stroke procedures to help address the SDoH affecting our patients. Our approach focuses on providing informed, targeted, and personalized health care for our patients by addressing their individual needs. Listening to our patients and learning how we can assist in their well-being before they leave the hospital will help with their post-stroke recovery, and also serve as a bridge between Crouse Hospital and the area we serve. We believe our findings and strategies can provide a framework for other small hospitals to likewise focus on SDoH to improve stroke outcomes in their communities.

## Methods

### Patient population

A retrospective analysis of patient information was conducted on the 1731 stroke incidents that were the focus of this study. The study began in February of 2021. The data were extracted from ED records, as well as laboratory analyses and health professional notes recorded electronically during the hospital stay of each patient starting from admission to the hospital until discharge. Additionally, electronic clinic records were accessed to determine if the patients came for a follow-up visit. Initially, we selected the charts of all patients discharged with a diagnosis of stroke (ischemic and hemorrhagic) or transient ischemic attack (TIA) from the Crouse Hospital ED from September 2015 to January 2021. This provided a starting pool of 4634 records to review. We then focused on a set of 1863 more complete records that included incidents from January 2019 to January 2021. Prior to January 2019, Crouse Hospital was gradually moving toward electronic data collection, so the data available from September 2015 to December 2019 was incomplete for the metrics we chose to examine. We did not have access to paper records stored in the hospital to complete this data set. This group was further reduced to those which were associated with home addresses in the six counties surrounding Crouse Hospital (Onondaga, Oneida, Oswego, Madison, Cayuga and Cortland). The final group of incidents used for our study numbered 1731. The authors involved in data extraction had access to patient identifying information during data collection. All identifiers were removed after data collection and prior to data analysis.

### Social determinants

The data collected provided a wealth of information on the care of the patients. To organize our study of the SDoH we classified our findings into four categories: demographics (including data points on age, sex, race, and geographical location of residence), health care access, institutional care, and discharge disposition. The focus of our assessment was on health care access–that being whether the patients lived in served or underserved areas. To operationalize service areas, we used the measurement provided by the United States Health Resources and Services Administration (HRSA) referred to as a Health Professional Service Area (HPSA), which assigns a score based on the home address of the patient [24]. The scoring method involves the tabulation of multiple metrics including: the Population-to-Provider ratio, the percent of the population in the area below the 100% Federal Poverty Level, the Infant Health Index (involving the Infant Mortality Rate or Low Birth Weight Rate), and the travel time to the Nearest Source of Care outside the HRSA designation area. A score on the HRSA scale indicates that the area is considered a priority for physician placement and additional Medicare reimbursements–a HPSA (an underserved area for our study).

Another consideration under our health care access category included health insurance. For this feature we examined whether the patients had government-sponsored insurance (Medicaid or Medicare), commercial insurance, or used other ways of paying for their medical care (Other; including self-pay, Workman’s Compensation, No Fault and Veteran’s Affairs). And to operationalize health care access, we examined whether the patients had a primary care provider (PCP). Having a PCP is described in the medical records as having a Medical Doctor, specialist, Doctor of Osteopathic Medicine, Nurse Practitioner, or Physical Assistant who is responsible for their care. If the patient reported having any of these health care professionals as their primary care provider, their records indicate that they have a PCP.

Part of our assessment of institutional care involved an analysis of exams that were administered in the ED. Certain evaluations are standardized such as the National Institutes of Health Stroke Scale (NIHSS), while others, such as a toxicology/drug screen, are up to the discretion of the provider–an important consideration in SDoH. The NIHSS is an instrument used by healthcare providers to objectively quantify the neurological impairment caused by a stroke. Along with other determinations such as the last known well (when they were last at their baseline or when they last felt normal), and imaging results, the NIHSS metric is a part of the patient assessment used to determine the type of immediate intervention necessary, and the post-acute care disposition of the patient [25]. With a score of 0 considered normal, the higher the NIHSS score the worse neurological deficit of the patient (the highest possible score is 42). Along with the NIHSS, we also recorded if the patient received a thrombolytic as part of their stroke care. Then to assess the severity of patient disposition we recorded the number of days the patient remained in the hospital. Our final metric for institutional care tracked whether the patient came in for a follow-up visit after discharge.

To gain an understanding of the state of health of the patients following their time in the hospital, we surveyed their discharge disposition as our final SDoH metric. We categorized the various levels of care at discharge as discharged to another facility, discharged home with or without help, discharged to hospice care and if the patient expired at the hospital.

### Statistical analysis

For our analyses, we first provided descriptive investigation of all the variables. Then, we studied the cross-sectional association between our focal variables and service areas. We analyzed demographic data, health care access, institutional care and discharge disposition using the metrics described above. We constructed separate multivariable analyses for individuals living in underserved areas (HPSAs) compared with those living in served areas, and then repeated these analyses with race as our outcome variable by looking at differences between White and Black patients–the main racial groups identified in our study. We determined the significance of these associations using an analysis of variance of independent *t*-test/ANOVA for continuous variables and Pearson chi-square tests for categorical variables. The data were considered significant with a *p*-value of less than 0.05. The statistical analyses were conducted using either SAS (Version 9.4) or IBM SPSS Statistics (Version 28).

### Ethics statement

Data collection and procedures for this study were approved by the Crouse Hospital Institutional Review Board. Since this study involved a retrospective review of patient data and did not pose a risk to the health of the patients, informed consent did not apply. To protect the personal information of the patients, the data file was stripped of identifiers prior to analysis.

## Results

### Descriptive analysis

Most patients in our study were White, (87.5%), while the rest of the incidents corresponded to patients identifying as Black (9.9%), Hispanic (1.3%), Asian (0.5%), Indigenous (0.1%), or Other (0.8%; Table 1). This breakdown was compared to race data extracted from the 2020 US Census (Table 2) for the six counties surrounding Crouse Hospital. Since the number of patients identifying as Hispanic, Asian, Indigenous, and Other were few, when race was considered, we grouped them together and classified the race data as White, Black, and Other (2.6%; Table 1).

**Table 1:** Sample characteristics. Included are the general data collected on 1731 incidents of stroke from the Crouse Hospital emergency department between January of 2019 and January of 2021.

**DEMOGRAPHICS**	
***Race (%)***	
White	87.5
Black	9.9
Other	2.6
***Sex (%)***	
Female	51.5
Male	48.5
***Age*** *(mean)*	72.1
***Geographical Location (%)***	
Onondaga	69.6
Oswego	15.5
Madison	7.7
Other	7.2
**HEALTH CARE ACCESS**	
***Underserved Area (%)***	58.9
***PCP (%)***	92.6
***Insurance (%)***	
Medicaid	7.7
Medicare	68.3
Commercial	18.4
Other	5.7
**INSTITUTIONAL CARE**	
***Toxicology (%)***	5.9
***Thrombolysis (%)***	10.0
***NIHSS (mean)***	4.9
***Days in hospital (mean)***	4.2
***Follow-up visit (%)***	57.3
**DISCHARGE DISPOSITION (%)**	
Secondary facility	29.0
Home with help	18.3
Home	44.9
Expired	7.3
Hospice	0.5

**Table 2:** 2020 US census data of race breakdown reported for each county. Government census data on the population breakdown by race (%) for Onondaga county and those that surround it. Source: https://www.census.gov/quickfacts.

	Onondaga	Oswego	Madison	Cayuga	Cortland	Oneida
White	79.8	95.8	94.5	91.6	94.1	85.6
Black	12.0	1.2	2.0	4.6	2.2	7.2
Hispanic	5.5	2.9	2.5	3.3	3.1	6.6
Asian	3.8	0.7	0.9	0.7	1.1	4.2
Indigenous	1.0	0.5	0.8	0.5	0.4	0.4

The analysis of age, sex, and geographical location of residence indicated that the average overall age was 72.1, that our study population was 51.5% female and 48.5% male, and that about 70% of the stroke incidents corresponded to patients who were from the surrounding county of Onondaga (69.6%). Patients living in Oswego County accounted for 15.5% of the data, while patients residing in the other counties of Madison, Cayuga, Cortland and Oneida made up 7.7%, 3.2%, 2.4% and 1.6% of the data, respectively–with Cayuga, Cortland and Oneida grouped together as Other (7.2%; Table 1).

The health care status of the patients was assessed by classifying the level of service (served or underserved/HPSA), looking for evidence of a primary care provider (PCP), and recording the type of health insurance for each incident. As noted in Table 1, about 58.9% of the patients were underserved while 92.6% had a PCP. Most of the patients had Medicare coverage (68.3%) while those with commercial insurance, Medicaid and other forms of insurance represented 18.4%, 7.7%, and 5.7%, respectively.

In terms of institutional care, the average NIHSS score was 4.9. About 10.0% of patients received a thrombolytic injection, and 5.9%, were given a toxicology test. On average, stroke patients spent 4.2 days in the hospital and 57.3% of them had a follow-up visit (Table 1). Looking at discharge disposition (Table 1), we observed that 44.9% of patients were discharged home, 18.3% were sent home with help, 29.0% left for another facility, 0.5% were sent to hospice care, and 7.3% died at the hospital.

### Multivariate analysis

#### Selected analysis by HPSA

**Fig 1:**
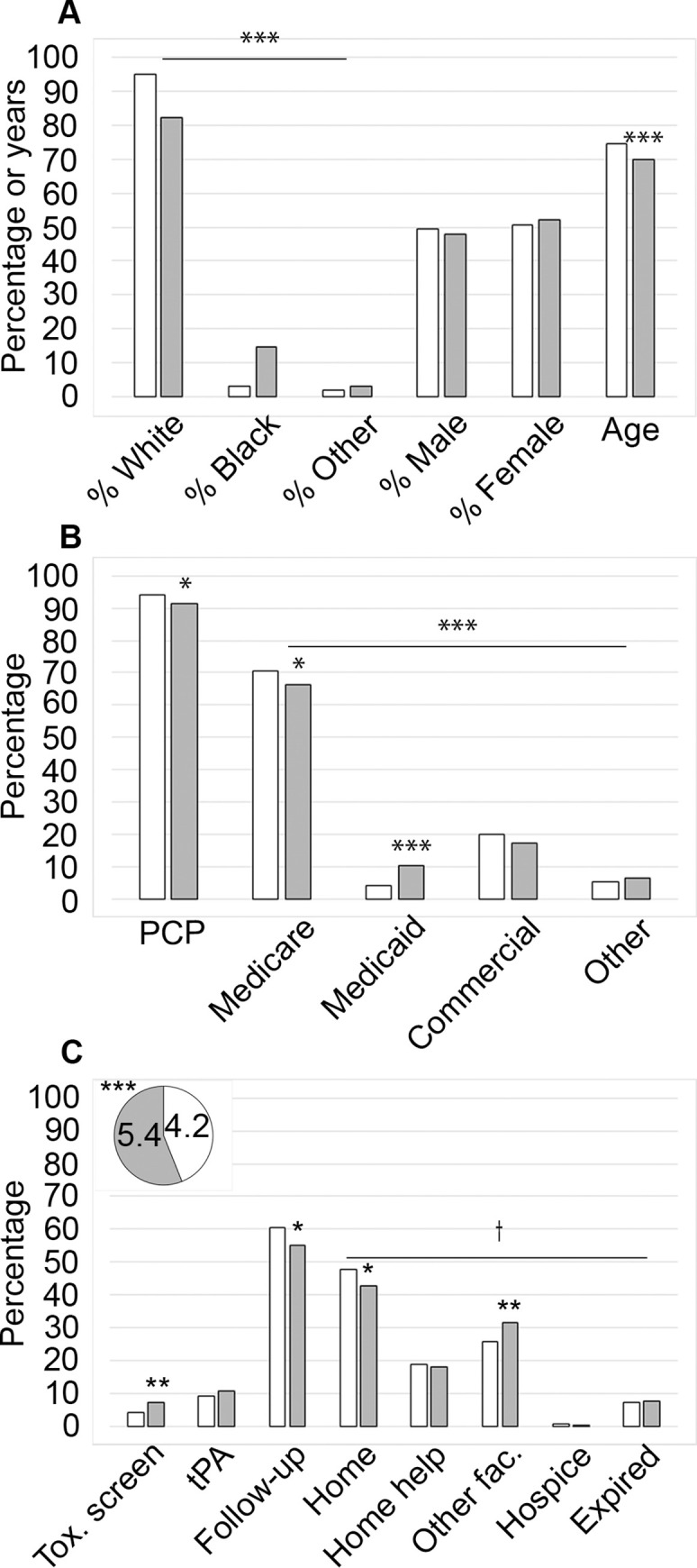
Comparison of patient demographics between served and underserved patient groups. A comparative analysis of served (white bars and pie piece; n = 712) and underserved (grey bars and pie piece; n = 1019) patients in terms of (A) demographics, (B) healthcare access and (C) institutional care and discharge disposition. The mean NIHSS values are shown in the pie chart inset. Symbols indicate significance at the following levels using an analysis of variance of independent *t*-test/ANOVA for continuous variables and Pearson chi-square tests for categorical variables.: †*p* ≤ 0.10, *p ≤ 0.05, **p ≤ 0.01, ***p ≤ 0.001.

*Demographic data*. Fig 1A illustrates the demographic breakdown of the patients by service. While most of the patients were classified as White for both served and underserved patients (94.9% and 82.2%, respectively), Black patients and those considered in the Other category tended to be underserved by population with 3.1% being in the served Black group, 14.7% in the underserved Black category, and 2.0% and 3.0% making up the served and underserved populations in the Other category, respectively. When we instead examined service by race (Table 3), 55.0% of White patients, 87.2% of Black patients and 68.9% of patients classified as Other were underserved, and the variance in the data was significant (*p* < 0.001). As for sex, from the underserved patients 52.2% were female and 47.8% were male. When compared to the data from served locations (Fig 1A) we observed that there was not a significant association between sex and service area (*p* = 0.5; Table 3). The mean age of the stroke patients who live in underserved areas was 70.2 compared to 74.8 (Fig 1A) for those who lived in served areas, which did result in a significant difference with *p* < 0.001.

**Table 3:** Bivariate relationships between service status and focal variables. Included are the data from the underserved population of patients that arrived at the Crouse Hospital emergency department with stroke symptoms between January 2019 and January 2021. n = 1019 incidents of 1731 total (compared to served patients, n = 712). Symbols indicate significance at the following levels using an analysis of variance of independent *t*-test/ANOVA for continuous variables and Pearson chi-square tests for categorical variables.: †*p* ≤ 0.10, *p ≤ 0.05, **p ≤ 0.01, ***p ≤ 0.001.

	Underserved	*p-*value
**DEMOGRAPHICS**		
***Race (%)***		0.000***
White	55.0	
Black	87.0	
Other	68.9	
***Sex (%)***		0.500
Female	52.2	
Male	47.8	
***Age (mean)***	70.2	0.001***
***Geographical Location (%)***		0.000***
Onondaga	43.0	
Oswego	97.0	
Madison	99.3	
Other	87.1	
**HEALTH CARE ACCESS**		
***PCP***	91.5	0.030*
***Insurance***		0.000***
Medicaid	10.1	0.000***
Medicare	66.1	0.018*
Commercial	17.3	
Other	6.5	
**INSTITUTIONAL CARE**		
***Toxicology (%)***	7.3	0.004**
***NIHSS (mean)***	5.4	0.000***
***Thrombolysis (%)***	10.7	0.799
***Days in hospital (mean)***	4.4	0.622
***Follow-up visit (%)***	55.0	0.018*
**DISCHARGE DISPOSITION (%)**		0.060†
Other facility	31.4	0.008**
Home with help	18.0	
Home	42.9	0.044*
Expired	7.5	
Hospice	0.3	

When we disaggregated the data by county of residence as noted on Table 3, there was a significant difference between the number of stroke patients who were from served and underserved areas in each county–with underserved patients from Onondaga making up 43.0% of that study population, while the underserved proportions from the other counties, which are predominantly rural, being 97.0%, 99.3% and 87.1% from Oswego, Madison and the other three counties together, respectively (*p* < 0.001).

*Health care access*. When looking at health care access by service status, we observed a significant variation between served and underserved patients. As demonstrated in Fig 1B, most served and underserved patients had a PCP (91.5% and 94.2% for the underserved and served, respectively), yet the difference between the groups was significant (*p* = 0.030; Table 3). There was a significant variation with type of health insurance and service area (*p* < 0.001; Table 3). Given the average age of the study population, Medicare was the most common form of insurance regardless of service (66.1% for underserved patients and 70.6% for those that were served; Fig 1B). Of those visits from individuals who were underserved, 17.3% had Commercial insurance, 10.1% had Medicaid and 6.5% were classified as having Other forms of payment. The underserved patient population was significantly more likely to be insured by Medicaid than served patients (10.1% versus 4.2% for underserved and served, respectively; *p* = 0.018; Table 3), and were less likely than the served patients to have Medicare (66.1% compared to 70.6% for underserved and served patients, respectively; *p* < 0.001; Table 3).

*Institutional care*. Our analysis of service status by institutional experience revealed four notable findings, as shown in Fig 1C. There were disparities in toxicology testing, NIHSS assessment (Fig 1C inset), follow-up care, and discharge disposition for the underserved population. First, of the 1731 records analyzed, 5.9% included a toxicology screening (Table 1). To learn more about this study population, we delved deeper into the circumstances of the patients involved. Looking at the incidents by service area, 3.9% of the served population were tested, while underserved people were analyzed almost twice as often (7.3%), and this difference was statistically significant (*p* = 0.004; Fig 1C and Table 3). Second, stroke patients from underserved areas had a statistically significant higher average NIHSS evaluation at 5.4, compared with those from served areas at 4.2 (*p* < 0.001; Fig 1C and Table 3).

Next, overall, 57.3% of the stroke patients did return to the clinic (Table 1) for a follow-up visit, while 7.3% of the patients died before being discharged, and 2.4% of the patients passed away shortly after discharge, before their follow-up visit. Therefore, 33.0% of patients coming to the ED for stroke did not come for a follow-up visit. Looking at this breakdown by service area (Fig 1C), 60.7% of served individuals, and 55.0% of underserved patients, had a follow-up visit which was significantly different between the two groups (*p* = 0.018; Table 3). We did not observe significant differences for the rate of thrombolytic treatment between served and underserved patients (9.1% and 10.7%, respectively), or for the average number of days in the hospital (3.99 versus 4.42 for the served or underserved, respectively).

*Discharge disposition*. Finally, we examined whether service status affected the discharge disposition of the patients (Table 3 and Fig 1C). Among the served cases, 47.9% left the hospital for home, while 18.8% went home but needed support, 25.6% of the served patients were discharged to another facility, 0.7% left for hospice care, and 7.0% expired in the hospital. The same analysis indicated that 42.9% of underserved patients were discharged home and 18.0% went home with help while 31.4% went to a secondary facility, 0.3% went to hospice and 7.5% expired. These variations were observed to be statistically significant overall between the two groups (*p* = 0.06). Furthermore, the differences between the groups indicated that served patients were significantly more likely to be sent home without needing help (*p* = 0.044), and that underserved patients were more likely to go to a secondary facility (*p* = 0.008).

### Selected analysis by race

**Fig 2:**
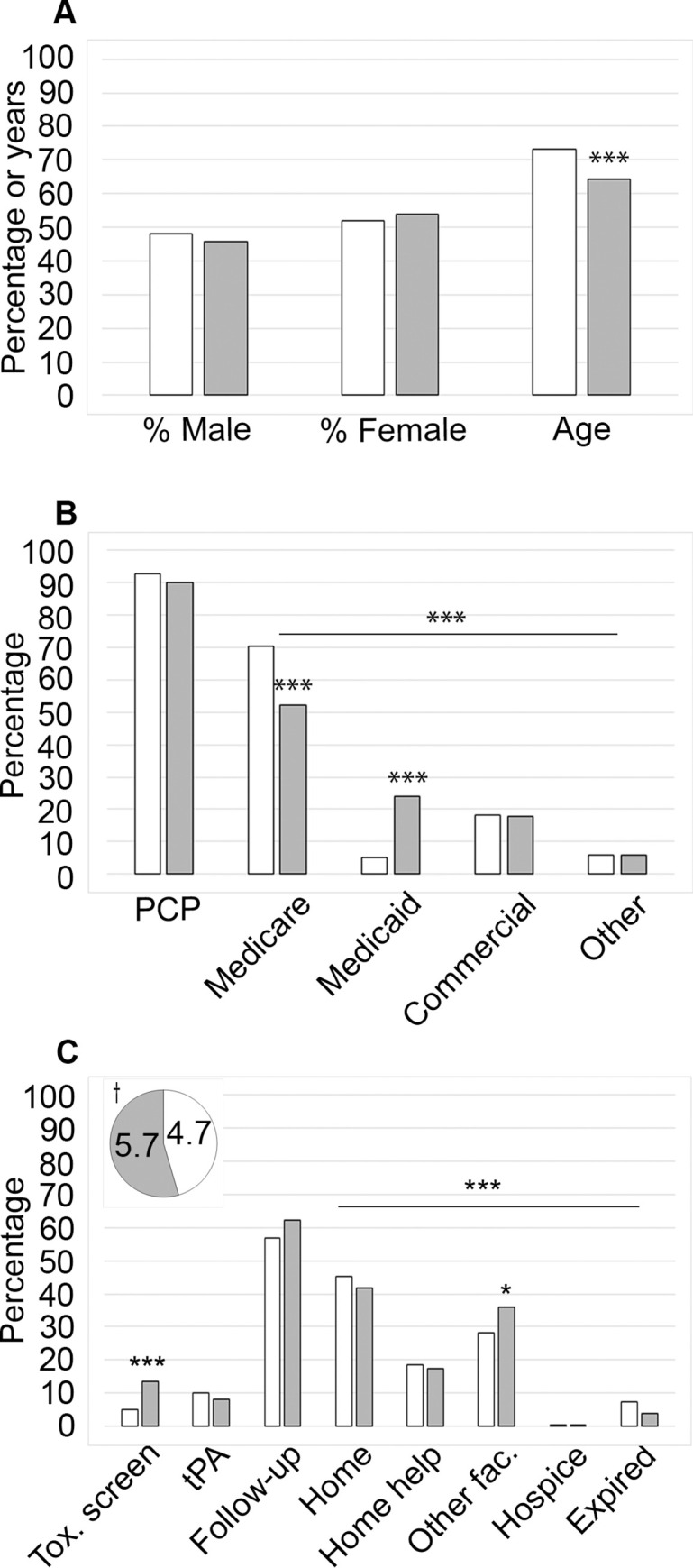
Comparison of patient demographics between White and Black patient groups. A comparative analysis of White (white bars and pie piece; n = 1514) and Black (grey bars and pie piece; n = 172) patients in terms of (A) demographics, (B) healthcare access and (C) institutional care and discharge disposition. The mean NIHSS values are shown in the pie chart inset. Symbols indicate significance at the following levels using an analysis of variance of independent *t*-test/ANOVA for continuous variables and Pearson chi-square tests for categorical variables.: †*p* ≤ 0.10, *p ≤ 0.05, **p ≤ 0.01, ***p ≤ 0.001.

*Demographic data*. Looking at age by race (Fig 2A), White patients presented at an average age of 73.1, while Black patients presented at an average age of 64.4, and the average age was 65.6 for the Other race category. This discrepancy in age indicates that racialized patients are having strokes at an earlier age (*p* < 0.001; Table 4).

**Table 4:** Bivariate relationships between race and focal variables. Included are the data from the Black population of patients that arrived at the Crouse Hospital emergency department with stroke symptoms between January 2019 and January 2021. n = 172 incidents of 1731 total (compared to White patients, n = 1514). Symbols indicate significance at the following levels using an analysis of variance of independent *t*-test/ANOVA for continuous variables and Pearson chi-square tests for categorical variables.: †*p* ≤ 0.10, *p ≤ 0.05, **p ≤ 0.01, ***p ≤ 0.001.

	Black	*p-*value
**DEMOGRAPHICS**		
***Sex (%)***		0.571
Female	54.1	
Male	45.9	
***Age (mean)***	64.4	0.000***
**HEALTH CARE ACCESS**		
***PCP***	90.1	0.218
***Insurance***		0.000***
Medicaid	23.8	0.000***
Medicare	52.3	0.000***
Commercial	18.0	
Other	5.8	
**INSTITUTIONAL CARE**		
***Toxicology (%)***	13.4	0.000***
***NIHSS (mean)***	5.7	0.084†
***Thrombolysis (%)***	8.1	
***Follow-up visit (%)***	62.2	0.174
**DISCHARGE DISPOSITION (%)**		0.000***
Other facility	36.1	0.039*
Home with help	17.4	
Home	41.9	
Expired	4.1	
Hospice	0.6	

*Health care access*. We further examined health care access by race (Fig 2B) and observed that Black patients were less likely to have a PCP, less likely to have Medicare coverage, and more likely to be on Medicaid, compared to their White patient counterparts (Table 4). There was no statistically significant difference between White (92.7%) and Black (90.1%) patients, regarding their PCP status. Overall, there was a significant difference in the variation between White and Black patients in terms of insurance (*p* < 0.001; Table 4). More specifically, about 23.8% of Black patients had Medicaid coverage while 5.3% of White patients fell into this category, which was significantly different (*p* < 0.001; Table 4). Likewise, there was a significant difference (*p* < 0.001) between the number of Black patients with Medicare coverage (52.3%) compared to White patients (70.5%; Fig 2B; Table 4).

*Institutional care*. As for institutional care by race (Fig 2C), we observed differences in toxicology screening, NIHSS evaluations, and thrombolytic administration. While 4.9% of the White population of patients were included in toxicology screening, 13.4% of Black patients underwent this analysis, which was greater than 2.5 times that of the White patient population (*p* < 0.001). Our examination of the mean NIHSS score (Fig 2C inset) by race showed that Black stroke patients (5.7) had higher scores than the White population (4.7), a variation that was marginally significant (*p* = 0.084; Table 4). White patients (10.0%) were more likely to receive thrombolytic treatment, compared to their Black patient counterparts (8.1%), but the finding was not statistically significant (Table 4). Then, while the difference was also not significant, we observed that Black patients (62.2%) came into the clinic for a follow up visit at a higher rate than their White counterparts (56.8%; *p* = 0.174; Table 4).

*Discharge disposition*. As for discharge disposition we observed differences in overall trends between White and Black patients (Fig 2C; Table 4; *p* < 0.001). Black patients were significantly more likely to be sent to another facility (36.1%; *p* = 0.039), compared to White patients (28.2%). Other trends indicated that Black individuals were less likely to be sent home (41.9% when compared to White patients, 45.5%), home with help (17.4% versus 18.4% for White patients), or to expire (4.1% compared to 7.5% for White patients). Importantly, there was only one Black patient sent to hospice.

### Selected analysis by service and race

To better appreciate some of the results observed, we further analyzed how service data was affected by race (by comparing the data for White and Black patients). Given the low numbers of Black patients overall (172), and served Black patients specifically (22), the statistical analyses conducted on these metrics only offers to inform our findings of possible underlying trends to monitor (Table 5).

**Table 5:** Bivariate relationships between service status with race and focal variables. Included are the data from the served Black population of patients (n = 22), the underserved Black population (n = 150), the served White population (n = 676), and the underserved White population (n = 838) that arrived at the Crouse Hospital emergency department with stroke symptoms between January 2019 and January 2021. n = 1731 total. Symbols indicate significance at the following levels using Pearson chi-square tests for categorical variables.: †*p* ≤ 0.10, *p ≤ 0.05, **p ≤ 0.01, ***p ≤ 0.001. Categories without any patients were not included in the chi-square test as indicated by (0.0).

	Served Black	Underserved Black	Served White	Underserved White	*p-*value	
**DEMOGRAPHICS**						
***Age (mean)***	72.0	63.3	75.0	71.7		
**HEALTH CARE ACCESS (% of race by service)**						
***PCP***	95.5	89.3	94.1	91.7	0.125	
***Insurance***						
Medicaid	4.5	26.7	4.0	6.3	0.000***	
**INSTITUTIONAL CARE (% of race by service)**						
***Toxicology (%)***	9.1	14.0	3.7	5.9	0.000***	
***Follow-up visit***	72.7	60.7	60.5	53.8	0.020*	
**DISCHARGE DISPOSITION (% of race by service)**						
Other facility	22.7	38.0	25.6	30.3		0.013*
Home with/without help	72.7	57.3	66.9	61.5		0.045*
Expired	(0.0)	4.7	7.0	7.9		0.355
Hospice	4.6	(0.0)	0.6	0.4		0.025*

*Demographic data*. We observed that the average patient age for served patients was higher for both White and Black patients but determined that the age of served Black patients was comparable to that of both served and underserved White patients (over 70 years of age), while that for underserved Black patients was 63.3 (Table 5).

*Health care access*. Examining health care access by service and race, we observed that while the numbers were similar, the trend remained that served individuals reported having a PCP more frequently than underserved individuals regardless of race, but that served Black patients reported having a PCP more than the other groups (with only one patient in this group not having a PCP), while underserved Black patients had the lowest percentage (95.5%, 89.3%, 94.1% and 91.7% for the served Black, underserved Black, served White and underserved White groups, respectively; Table 5). Then, when comparing insurance coverage, we examined the numbers with reference to patients covered by Medicaid. For this analysis we determined that the underserved groups did report having Medicaid coverage more than that of served patients, and that this was particularly prevalent for the underserved Black population (26.7%), which was almost five times that of the underserved White population (6.3%). The served Black and served White groups reported having Medicaid coverage at a rate that was about 4% (4.5% and 4.0%, respectively; Table 5). These results were observed to be statistically significant (*p* < 0.001), however there was only one patient in the served Black category with Medicaid insurance.

*Institutional care*. We examined several aspects of our institutional care metrics by service and race. Assessing the toxicology screen, we determined that underserved patients were examined more than served, and that served Black patients (9.1%) were assessed less frequently than those that were underserved (14.0%). However, the fraction of both served and underserved Black patients exceeded that of served (3.7%) and underserved (5.9%) White patients (Table 5). The observed variation between the four groups was significant (*p* < 0.001) even though only two served Black patients were tested. Further assessing the follow-up rate (Table 5), we determined that served Black patients came for a follow-up appointment at the highest rate (72.7%; 16 incidents) followed by underserved Black patients (60.7%), served White patients (60.5%) and then underserved White patients (53.8%; *p* = 0.020).

*Discharge disposition*. We also endeavored to better understand the discharge disposition metric by looking at service and race. The underserved groups did go to another facility more frequently than served groups, with the Black underserved population having the highest percentage– 22.7%, 38.0%, 25.6%, and 30.3% for the served Black, underserved Black, served White and underserved White populations, respectively (*p* = 0.013; Table 5). Examining the numbers for the groups that were sent home–in this case with or without help grouped together–the served populations, and particularly the served Black population—were sent home more frequently (72.2% of served Black patients and 66.9% of served White patients) than the underserved patients with the underserved Black population being sent home with the lowest rate (57.3% of underserved Black patients and 61.5% of underserved White patients; *p* = 0.045; Table 5). Then focusing on the breakdown for those that expired or were sent to hospice, the significance of the data did not include served Black patients for those that expired, or the underserved Black population for those sent to hospice since there were no events in either category. Underserved Black patients had the lowest frequency of death (4.7%), when compared to served White (7.0%) and underserved White patients (7.9%; Table 5). Finally, of those sent to hospice (Table 5), the served Black patient group had the highest frequency in this category (4.6%), while the rate for served White and underserved White, was 0.6% and 0.4%, respectively. The data were analyzed as significant (*p* = 0.025), but there was only one served Black patient in the group.

## Discussion

The objective of this study was to identify the SDoH affecting stroke outcomes within the Crouse Hospital community to further our mandate of providing the best quality stroke care to our patients. Through our retrospective analysis of these factors, we observed several disparities associated with racialized patients and individuals residing in underserved areas. These findings provided our group–Crouse Neurosciences—with information to put in place strategies to assist these communities. We believe other centers can learn from our experience to further advance stroke care by considering SDoH with the lens of a small community hospital setting.

Most individuals from the counties surrounding Crouse Hospital are White, and most Black individuals reside within Onondaga county and in the city of Syracuse. Consequently, most of the patients coming to the Crouse Hospital ED with stroke symptoms were White followed by Black and then Hispanic identifying individuals (Fig 1A; Table 1). The remaining groups identified were Asian, Indigenous and Other (Other, Asian and Indigenous patients were grouped with Hispanic individuals into a broadened Other category due to low numbers). When comparing the observed demographic data with that of Onondaga county (Table 2), we determined that our patient population had a greater proportion of the White community and less for every other racialized group than the government provided demographic data would suggest. In contrast, compared to the surrounding counties, the incidents corresponding to White patients at Crouse Hospital was lower for the most part, while that of Black individuals was higher. Other racialized groups are still lower at Crouse Hospital. We are uncertain whether this discrepancy is because these populations choose to go to one of the other two major area hospitals or if other factors are preventing these groups from making it to the ED once stroke symptoms begin. By way of example, the proportion of persons of Asian (0.5%) background arriving at the hospital with a stroke diagnosis was considerably below the published population data (Tables 1 and 2). Since other studies demonstrated that Asian individuals had a lower level of stroke awareness compared to other racial groups, members of this community may not be aware of stroke symptoms and therefore do not present to the hospital setting—but it might also be that people identifying as Asian are choosing to go to a different hospital and that might have been what was responsible for the lower numbers [26].

When suspected stroke patients arrive in the ED, they undergo several health assessments to evaluate their neurological status and determine a care plan. One standardized test is the National Institutes of Health Stroke Scale (NIHSS) which is a quantitative (0–42), systematic, and validated measurement and is one tool, in addition to several other factors such as the determination of the last known well (the last time the patient reported feeling normal) and imaging results, used to determine if the ED should implement a “code B” or code brain at Crouse Hospital [27]. Higher numbers on the NIHSS indicate more severe stroke symptoms. Importantly, all strokes are given a NIHSS ranking even if a code B is not implemented. Once a code B has been determined, specific clinical criteria are evaluated to determine if stroke patients are candidates for intravenous thrombolytic treatment for acute ischemic stroke. For the duration of this retrospective study, Crouse Hospital used alteplase or (recombinant) tissue-type plasminogen activator (r-tPA) as the go to thrombolytic therapy, which has been demonstrated to improve stroke outcomes [28].

We observed that Black patients had higher NIHSS scores than those that were White, which may indicate differences in the general state of health between the two populations prior to the onset of their stroke (Fig 2C inset; Table 3). SDoH contributing to this idea may include access to a PCP and the type of health insurance held by the patient. While our data did indicate that most of our patients had a PCP (Table 1) we did observe that underserved and Black patients reported having a PCP at a lower rate than White patients (Figs 1B and 2B, Tables 3 and 4) and had a higher probability of having Medicaid coverage instead of Medicare, which was more commonly held by White and served individuals (Figs 1B and 2B; Tables 3 and 4). Referring to the Black population, the difference in coverage may be attributed to the younger average age at which Black patients arrived at the hospital with stroke symptoms, and the same may be true for the subset of White patients with Medicaid (Fig 2A; Table 4). Medicaid coverage has fewer provisions for stroke treatment than Medicare, however, given that working age individuals, particularly those that are non-White, are comprising an increasing share of stroke patients, this may change in future [20, 29].

Furthermore, even though the low numbers of served Black individuals (22 total) precluded a clear assessment of significance, we did observe that underserved White and Black patients both reported having a PCP less frequently than served patients of both racial groups, with only one served Black patient reporting that they did not have a PCP (Table 5). However, underserved Black patients did have the lowest probability of having a PCP compared to the other groups. And, in terms of Medicaid coverage, despite served patients of both races having about the same percentage of individuals with Medicaid coverage (Table 5), which was less than that of the underserved groups, almost five times more underserved Black patients had Medicaid coverage compared to underserved White patients. This variation was significant despite the low number of served Black patients included in our study (only one of 22 served Black patients had Medicaid coverage; Table 5). While more than 90% of patients covered by commercial insurance or Medicare reported having a PCP, only 78.2% of those with Medicaid reported the same, reinforcing the idea that those on Medicaid are likely not receiving regular primary care.

However, even though Black individuals and the underserved were observed to have a PCP with a slightly lower frequency than served or White patients the data indicate that most patients did report having a PCP. That said, what the data do not consider was the type of provider seen (specialist physician, Internist, or nurse practitioner for example) or how regularly patients connected with these individuals to monitor their general state of health and their adherence to medication regimens to treat underlying conditions (Figs 1A and 2A). While not required at Crouse Hospital, some of the records did specify if the patient identified a physician or other professional as their PCP. From these data we observed that some patients listed a specialist (such as a cardiologist) as their PCP; and at least 4.5% of our patients were recorded as having a nurse as point of contact for primary care—and of that group, 17% were Black.

Furthermore, when we endeavored to better understand how service plays in the average age difference between Black and White patients (Fig 2A and Table 4) despite the relatively low number of served Black individuals, we observed that served White and Black patients, as well as underserved White patients have an average age of stroke that is more comparable when contrasted to underserved Black individuals (Table 5). This finding suggests that access to primary care has the potential to improve the situation for racialized individuals but may not have as much of an effect on the White population. Given this observation, and since racialized and underserved patient groups are reported in the literature to have less PCP coverage and instead use the ED when they need medical assistance, it will be important for us to gauge if our patients have a PCP–paying particular attention to the underserved and members of racialized groups [29]. As we gain a better understanding of the primary care situation of the patients in our care, we will be able to take steps to mediate coverage for these patients.

While the rate of thrombolytic administration (tPA) between served and underserved groups was similar, we did observe that White patients were more likely to receive tPA to treat their stroke compared to those that were Black (Fig 2C; Table 3). While the difference was not statistically significant, it does point to a trend that we plan to further evaluate. TPA may be provided to patients who have had an ischemic stroke within a certain time window since they were at their baseline (last known well). The observation that Black patients received tPA with a lower frequency than White patients may indicate that Black individuals arrive in the ED with a last known well that places them outside of the time window for thrombolytic treatment (at most 4.5 hours) with a greater frequency than White patients, or that Black individuals are presenting with more hemorrhagic strokes which are not amenable to tPA treatment, as opposed to ischemic strokes which are. Since this issue can be informative to patient stroke education in that people might not realize that there is a treatment window for certain kinds of strokes that can make real impact on stroke recovery—we plan to carefully document this data among our patients moving forward and include the information in our community outreach efforts.

In addition to the standard examinations of NIHSS and an assessment for the use of thrombolytics, some stroke patients are administered a toxicology test to detect illicit drug use. The screen involves a urinalysis for amphetamines, barbiturates, benzodiazepine, cannabinoids, cocaine, opiates, and phencyclidine. Some researchers have documented that non-White patients and pregnant women are more likely to be screened for drug use than the general population [30, 31]. On this front, we observed that the underserved and Black patients more frequently underwent a toxicological screen (Figs 1B and 2B; Tables 3 and 4). Furthermore, when service level and race were considered, the variation was significant. We determined that Black served patients had a drug screen more frequently than White served patients (Table 5) and further observed that this trend extended for the underserved where Black individuals were also tested at a higher rate than those that were underserved and White (Table 5). This test may have been done because the use of cocaine and amphetamines has been associated with an increased risk of stroke but since this test is up to the discretion of the provider we do not know why the test might have been ordered [32]. Significantly, the toxicological test is one of several analyses that are conducted if a patient arrives to the ED with an “altered mental state”–including assessments for seizure and for an active urinary tract infection (UTI)–to help determine if a patient is suffering from a stroke or some other issue. Since each of these conditions invoke different protocols the addition of a toxicological screen in these cases is necessary to allow for the appropriate treatment. Furthermore, it is important to consider that our findings may also reflect the increasing disparity wherein racialized individuals are dealing with addiction, but are suffering without the same access to treatment available to White individuals–underscoring the need for effective primary care [33–35]. Assessing the underlying reason for the observed trend will require a greater patient sample size to better appreciate the meaning of the results, an analysis of the circumstances (stroke and non-stroke) of all the toxicological screens in the ED, and a deeper dive into provider notes prior to the test.

The number of days admitted to the hospital and the frequency of in-clinic follow-up visits are two other variables we examined to assess the institutional care experience of our patients. Nationally, stroke patients are typically admitted to the hospital for three to seven days [36]. The average length of stay observed in our data was within this national average, and while the underserved patients did have slightly longer average stays, the differences were not significant (Tables 1 and 3). Patients admitted for longer periods might have presented with worse stroke symptoms and thus required a more extensive hospital stay for their stroke care. The reasons underlying these extended stays may be reflective of the same factors–insurance and PCP-related issues–as we discussed for our NIHSS analysis above.

As for follow-up visits, it is a priority for Crouse Neurosciences that patients who were treated in the ED for stroke be scheduled for an in-clinic visit within approximately 30 days after discharge–primarily through an appointment with the Neurology department, but also with the Neurosurgery department if a surgical procedure took place as a stroke intervention. If a patient had an appointment with another clinic within the Crouse network such as Cardiology, or General Practice–they were still considered to not have had a follow-up visit. The reason for this was two-fold; the first being that other departments are not focused on stroke care in the same way as the providers in the Neuroscience departments, and secondly, since we are aiming to implement an interventional study, we needed to determine how effectively Crouse Neurosciences was able to bring our patients back for a follow-up visit. While we determined that 2.4% of our patients died after leaving the hospital before they could return to the clinic, we observed that only 53.7% of the patient charts included in this study, recorded evidence of a follow-up appointment (Table 1). Our analysis revealed that most of our patients come from an underserved area (Table 1) and that there is a significant disparity between the rate of follow-up between served and underserved patients (Fig 1C and Table 3). Interestingly, when comparing the data by race, we observed that Black patients were more likely than White patients to return for a scheduled appointment in the clinic (Fig 2C and Table 4). We delved deeper into this unexpected finding by examining the correlation between race and service and discovered that served Black patients came for a follow-up visit more frequently than their served White counterparts (Table 5). Conversely, underserved White patients attended a follow-up visit at a higher rate than underserved Black patients (Table 5). While it is difficult to interpret the underlying reason for these results because of the low number of served Black patients, the data were statistically significant, and do present a compelling trend. There are several reasons that might contribute to this observation. One may stem from the fact, as discussed above, Black patients tend to have a more severe stroke (based on NIHSS data) and therefore may be more inclined to present for follow-up care with our stroke team to treat these more serious symptoms. In addition, Black patients may have a PCP that may lack the expertise necessary to treat patients following a stroke, so they prefer to continue their care with our team. Alternatively, since the served Black patients are coming back for their appointments 12% more frequently, it may be that with being served and having the appointment scheduled with a known provider and being contacted by our staff for an office visit, may provide an added incentive to which Black patients respond better than White individuals. Others observed that Black patients will indeed respond on par with their White counterparts for a follow-up visit if suitably accommodated [37]. However, it may also be the case that served White individuals live further away from Crouse Hospital and prefer to follow-up with a provider closer to home, while served Black patients may reside right in Syracuse, so a visit to the clinic would comparatively be less of a burden. Since post stroke care is crucial for a better outcomes we will need to question patients who do not return for a follow-up visit to better understand what we can do to motivate more patients to come to the clinic following their discharge from the hospital [38].

The final SDoH metric we examined was the discharge disposition of the patients. Ultimately, this measurement was largely dependent on the severity and outcome of the stroke event, however this data point is not only affected by the underlying state of health of the patient as discussed above for the NIHSS analysis, but can also be a reflection of the local/home support network available to the patient and their type of insurance coverage [39–43]. We classified the discharge disposition (or destination) for our study as: home, home with help, other facility, hospice or expired. (Table 1). Our analysis indicated that there were significant overall variations in discharge disposition by both service level (Fig 1C and Table 3) and race (Fig 2C and Table 4). Specifically, we determined that the underserved were more likely to go to a different facility and less likely to be sent home compared to the served population. The trend also suggested that the underserved went home with help or to hospice at a lower rate than served patients, while the proportion of patients who expired was comparable between the two groups. The main types of secondary facilities considered were either for rehabilitation or for nursing care. Assessing the other facility data for served and underserved patients into these categories provided further information in that the underserved were more likely to be sent to a rehabilitation center (14.4% of underserved versus 7.9% of served patients) and less likely to go to a nursing home (3.3% of underserved versus 16.7% of served patients). Again, the type of insurance and age of the patients might have been factors influencing this observation, more than the severity of the stroke on admission given that the average NIHSS score was higher for the underserved patients. The underserved may also have fewer local supports if sent home, further influencing the observed higher rate of admission to a rehabilitation facility.

When comparing the outcomes between White and Black patients, as with the underserved analysis, Black patients were significantly more likely to be sent to another facility (Fig 2C and Table 4). The other discharge data trends also suggested that Black patients had a lower rate of being sent home, or home with help. In this case, however, Black patients, as with the underserved data above, were more frequently sent to a rehabilitation facility than White patients (11.6% and 9.7%, respectively), but unlike the underserved data, Black patients were also more likely to be sent to a nursing home (23.4% for Black patients and 17.6% for White patients). Unlike what we observed for the underserved data, however, the higher average NIHSS score may have influenced the rate at which Black patients required nursing care before being able to go home. We would have to go deeper into the discharge notes of these patients to better understand the variations in the data between the underserved and Black patient groups. Also, as discussed above for the underserved overall, Black patients, residing primarily in underserved areas (Table 3), might have less access to support systems that allowed them to go home compared to White individuals.

When considering the other aspects of the discharge disposition, while the proportion of Black patients sent to hospice exceeded that of White patients, it is important to note that these numbers reflect only one Black patient and eight White individuals. We would require an even larger sample size to better appreciate the rate at which patients are sent to hospice. We also determined that fewer Black individuals expired than those in the White category, despite the higher average NIHSS score metric for Black patients on admission as described above (Fig 2C and Table 4). It is also worth acknowledging again that the younger on average Black stroke population (Fig 2A and Table 4) might have supported a better recovery, and the shifted dynamics in health coverage with Medicaid being more common in this group (Fig 2B and Table 4) may also have been a factor in the destination of the patients due to differing forms of post stroke benefits offered by Medicaid when compared to Medicare.

Due to the importance of support systems in addition to access to primary care following discharge, we sought to study the discharge data further by examining how both race and service level affected the outcomes (Table 5). Again, the low number of served Black patients was a confounding factor in this combined analysis with some categories not having any patients and so could not be included in the statistical analyses (Table 5). Nevertheless, the analysis did reveal notable trends for us to consider. We observed that served White and Black patients did go home (with or without help) at comparable rates, and that while this proportion was lower for both underserved White patients and underserved Black patients, underserved Black patients were sent home less frequently (Table 5). These variations did show significance despite the sample sizes. Furthermore, combining the race and service analysis we determined that underserved Black individuals were the group most frequently sent to another facility, even when compared to the underserved White demographic with the other facility variations also showing significant differences between the four categories (Table 5). We also observed that served patients, and served Black patients, accessed hospice care more often than underserved patients–with none of our underserved Black patients being sent for hospice care (Table 5). Importantly, while significant, this data only involved one served Black patient being sent to hospice from our hospital. This is an interesting result and is in line with the recent finding that Black patients use hospice care less frequently than others [44]. Finally, when considering the number of patients expired, the rate for underserved Black patients was considerably lower than that of both served and underserved White patients which may reflect the younger average age of stroke and therefore a better recovery despite the higher initial NIHSS score data (Tables 3 and 5). In future we should also record the NIHSS data at discharge to get a better idea of how the stroke symptoms evolved while in the hospital. Altogether, while there are many caveats to consider, these findings do reinforce our sentiment that access to primary care is of utmost importance for more favorable post stroke outcomes.

Despite the general decrease in stroke incidence that has been achieved through the proactive treatment of underlying health issues associated with an increased risk of stroke in high income countries, the incidence of, and mortality due to stroke in disadvantaged populations has not changed or has even increased by some accounts [5, 45]. This unfortunate impact has lingered in the United States for decades without improvement in medically underserved populations. In fact, according to a report issued by the CDC in 2022, the rate of stroke in the Hispanic population has increased, the risk of stroke is twice as high for Black individuals as it is for those that are White, and the Black population has the highest rate of death due to stroke [1]. The American Heart Association/American Stroke Association, National Institutes of Neurological Disorders and Stroke, and the World Stroke Organization have committed to scale up efforts past the data collection phase, and meaningfully target these groups to make a change in the statistics [38, 46–48]. These organizations also agree that work needs to be done on understanding the reasons underlying the causes of these disparities, stating that clinical trials specifically take steps to address the disparity data collected, and that outreach to these communities needs to be undertaken in non-traditional settings (churches and barbershops for example). Furthermore, a concerted effort to use mobile health technology, and effective interventions by non-physicians to reach disparity groups needs to be made to ensure these members of society do not continue to be left behind [49–51]. Experts have even suggested that priority should be given to culturally tailored interventions to decrease the incidence of recurrent stroke in underserved populations [7].

Crouse Hospital is situated in the city of Syracuse in Onondaga county. Onondaga is a large county of almost 800 square miles (according to U.S. census data) including not only the city of Syracuse, but also an extensive rural area. The counties surrounding Onondaga are mostly rural. Therefore, it is important for us to also acknowledge that the greater the distance a patient must travel to a stroke center the chances of a poorer outcome increase because this demographic may need more support after discharge–and that the needed support in rural areas is likely lacking. As such, our plans must also consider how far patients must travel for their follow-up appointment, and how that might be affected by weather during the winter months. On this front, our outreach plans should also include efforts to provide alternative opportunities such as regular telemedicine visits for our most remote demographic to have a continuity of care [52, 53].

Moving forward, it is also important to assess our strategy in terms of how others have addressed similar concerns so we can benefit from their experience. While other studies used medical determinants to assess progress, research focused on these methods alone were not successful in meaningfully reducing stroke incidence in their study populations [9–11]. Studies aiming to work with social determinants, on the other hand, did report some success [5, 8]. The methods used in these reports included helping patients to adhere to care plans, mediating the transition of care after leaving the hospital, initiating stroke education and/or increasing physical activity along with rehabilitation. Considerable success was made when helping underserved patients adhere to their personal plan of care with a cultural and educational focus. The strategic use of technology such as health monitoring apps and text messages can provide a level of care and outreach to rural populations or to individuals that have difficulty accessing the clinic. The ASPIRE project recorded some success by focusing on addressing social determinants to reach more favorable clinical outcomes. A component of this study was community outreach with culturally tailored education materials and advertising. The researchers running the study assessed their progress by recording shorter arrival times and an improved rate of tPA administration [54].

From the outset of our study, we worked with the idea that those patients from HPSAs, or those who are medically underserved with respect to primary care, would likely have different stroke outcomes from those patients who were not. Others investigated if this factor alone–that being having less access to primary care by HPSA designation–was associated with reduced cardiovascular care and increased stroke [4]. These investigators determined that HPSA status alone could not account for the discrepancies they observed in their study population and concluded that HPSA status along with other social factors should be studied to improve clinical outcomes. Levine and colleagues [46], studied the disparities confronting non-White individuals in terms of stroke through a survey of clinical trials hoping to shed light on the issue and provided some insight into what approaches should be taken. They concluded that patient-centered, culturally tailored interventions should be a focus. Moving forward therefore, we need to keep in mind that treating underlying conditions during follow-up appointments is only part of the equation. Indeed, our findings support the idea that access to primary care providers and inclusive healthcare (supportive of stroke recovery) is important. However, based on the work of others, without a patient-centered and culturally sensitive approach considering the SDoH that may contribute to disparities in stroke care, we will likely only have limited success [8, 46].

Our retrospective analysis of in-hospital data relying only on documentation from health care providers lacks the patient perspective to understand their experience with stroke care, as well as the lived situations that occurred prior to the stroke event. Additionally, it does not provide insight into the out of hospital stroke statistics in our community, which would help us understand facets of the basic demographics collected (Table 1). Crouse Hospital is situated in the core of Syracuse near two other hospitals–one being a major state university hospital, which is located on the same city block, and the other a non-profit hospital within the Catholic Health System, which is situated within three city blocks. Therefore, while many analyses might not show discrepancies by service area and race–or conversely, provide differences despite low patient numbers in certain categories—our findings may not be reflective of the overall stroke statistics for the region since some patients may not have come to Crouse Hospital due to a preference for one of the neighboring hospitals. We also cannot account for those individuals who did not seek medical care for their stroke symptoms or gauge how many patients may have succumbed to a stroke without making it to the hospital for care. Of these drawbacks, we can work to address the first–in that we can focus on instituting the means to gather a more complete patient history to gain a greater understanding of how the SDoH pertaining to stroke care affects our patients, and thereby better understand what is happening within the community.

## Conclusions

This study provided us with critical insights into how SDoH affected the patients who came to the Crouse Hospital ED to access our comprehensive care for their stroke symptoms. We can use what we learned to develop targeted patient and community-centered action plans for stroke outreach and education and to gather information on what these different groups might need. Some tactics could include helping patients arrange for transport to their appointments, having flexible appointments for more remote patients, sending text reminders for appointments, using telehealth (particularly for racialized, immobile and remote patients), stroke education, encouraging participation in stroke survivor programs (the Crouse stroke survivor patient group meets monthly), and mitigating concerns preventing patients from adhering to their medication regimen, including perceived discrimination, inadequate continuity of care and the long-term effects of the medications. As a small community hospital, however, we must look at all these options to determine how we can have the greatest impact given limited staff and resources. In the final analysis, one theme dominated–access to a continuity of care can make a difference to stroke outcomes—so this is where we will focus our efforts. Using tailored questions for stroke patients upon discharge from the ED, we can assess the personal situations of the patients and work with them to develop strategies to increase the success of their post-stroke recovery. Specifically, by gathering information on their primary care provider we can inform that individual of the discharge status of the patient and mediate a follow-up visit with them, to complement our own in clinic visit. We can also assess how frequently the patient visited their PCP and provide education on the importance of regular visits with that individual. Where possible, we plan to look for partners within the Crouse network of primary care providers to assist us in delivering a coordinated care approach–a strategy that will be particularly useful for those without a primary care provider. Then, for those individuals who do not come in for a follow-up appointment with our providers, we will reach out to them to determine what is preventing them from connecting with us and develop a strategy, such as a telehealth visit, to provide a continuity of care for their neurological well-being. In the setting of the follow-up visit, we can inquire again regarding their primary care status to ensure that underlying health conditions that contributed to the stroke are being managed. These strategies are ones that can be adopted by other small community hospitals also looking for practical solutions to address the effects of the SDoH to improve the post stroke recovery of their patients. Ultimately, we hope that our efforts to address the complications imposed by the SDoH–specifically by improving the primary care situation for all our patients as a first approach, will increase the success of stroke recovery in the community and encourage others to come to Crouse Hospital for stroke care.

## Action plan

Our findings highlight how the establishment of regular primary and follow-up health care with a medical professional can positively impact the underlying health of individuals and help address disparities introduced by the SDoH affecting our community. These findings speak broadly about universal access to reliable, trusted primary care, and how, once in place, disparities can start to be addressed. At issue though, as we determined by studying our patient records, standard information collected on a patient in a typical ED visit does not provide a complete picture of patient needs, or how the SDoH affect them. To move forward then, the medical community should implement strategies to determine the specific needs of the patient and tailor a care plan that will support those requirements. The approach should consider the resource and staffing limitations of the institution. Leading by example, as a first step, the Crouse Hospital Neuroscience team is implementing a strategy wherein the in-office (clinic) nurse will go to the hospital to meet with each patient individually prior to their discharge. This plan provides the patient with a face to the name of the person who will reach out to them to schedule their follow-up appointment. At the same time, this clinic nurse can discover the needs of the patient, reinforce the importance of regular visits with a primary care provider, and work to address any concerns. In this way, our team will connect the personalized care of the in-hospital setting with that of the clinic, encouraging the patient to pursue continued care. We hope that other community hospitals will enact strategies such as this to begin to break the barriers to healthcare brought about by the social determinants of health to benefit the care of all patients.
